# Impact of the COVID-19 pandemic on the human resources for health in India and key policy areas to build a resilient health workforce

**DOI:** 10.12688/gatesopenres.13196.1

**Published:** 2020-10-15

**Authors:** Ankita Mukherjee, Rakesh Parashar

**Affiliations:** 1 Oxford Policy Management, New Delhi, 110049, India

**Keywords:** health systems, resilience, health crisis, COVID-19, human resource for health, health workers, policy framework, health resilience framework

## Abstract

The COVID-19 pandemic has disrupted the already low resourced, fragmented and largely unregulated health systems in countries like India. It has only further exacerbated the stress on human resources for health (HRH) in many unanticipated ways. We explored the effect of COVID-19 pandemic on the health workforce in India, and analytically extrapolated the learnings to draw critical components to be addressed in the HRH policies, which can further be used to develop a detailed ‘health workforce resilience’ policy. We examined the existing literature and media reports published during the pandemic period, covering the gaps and challenges that impeded the performance of the health workers. Recommendations were designed by studying the learnings from various measures taken within India and in some other countries. We identified seven key areas that could be leveraged and improved for strengthening resilience among the health workforce. The system-level factors (at macro level) include developing a health workforce resilience policy, planning and funding for emergency preparedness, stakeholder engagement and incentivization mechanisms; the organization-level factors (meso level) include identifying HRH bench strength, mobilizing the health workforce, psycho-social support, protection from disease; and the individual-level factors (micro level) include measures around self-care by health workers. In keeping with the interdisciplinary nature of the associated factors, we emphasize on developing a future-ready health workforce using a multi-sectoral approach for building its strength and resilience.

## Disclaimer

*The views expressed in this article are those of the author(s). Publication in Gates Open Research does not imply endorsement by the Gates Foundation.*

## Background

It is likely that most low and middle-income countries would fail to meet the health workforce requirements for providing advanced preventive and curative healthcare to all their citizens in the near future. The COVID-19 pandemic has once again highlighted the dismal reality of our health systems globally, and it calls for a paradigm shift in the health systems resourcing and policies. Although COVID-19 is being prioritised and all resources are directed towards its mitigation, it is also crucial to appreciate the burden of other acute and chronic illnesses, that are being neglected, for which we would require our ‘human resources for health’ (HRH) in its best armour.

India, with a total number of COVID-19 confirmed cases of 6.15 million as of 29 ^th^ September 2020, has recorded nearly 96,000 deaths ^1^. India’s weak ‘human resources for health’ (HRH) capacity and its inadequate deployment strategies could have been major contributors to the poor containment of the spread of the pandemic, in the country. The HRH includes all clinical, management and support staff related to health service delivery. Although there has been substantial growth in the number of health workers recently in India, the health workforce remains chronically insufficient (in terms of sheer numbers available as well as the skills required) in the public sector and is often irrationally distributed ^2^. Health emergencies and disasters would keep throwing the health workforce in stress. The numerous challenges faced by HRH eventually affect the health service delivery and population health outcomes. This is certainly not the last pandemic ^3^ we would witness; but, it is important to continuously learn from it and prepare adaptive health systems that function effectively during and post crisis. It is hence critical to understand the key challenges faced by HRH during a pandemic and develop a policy to ensure the resilience of health workforce during any future catastrophes.

**Figure 1.**
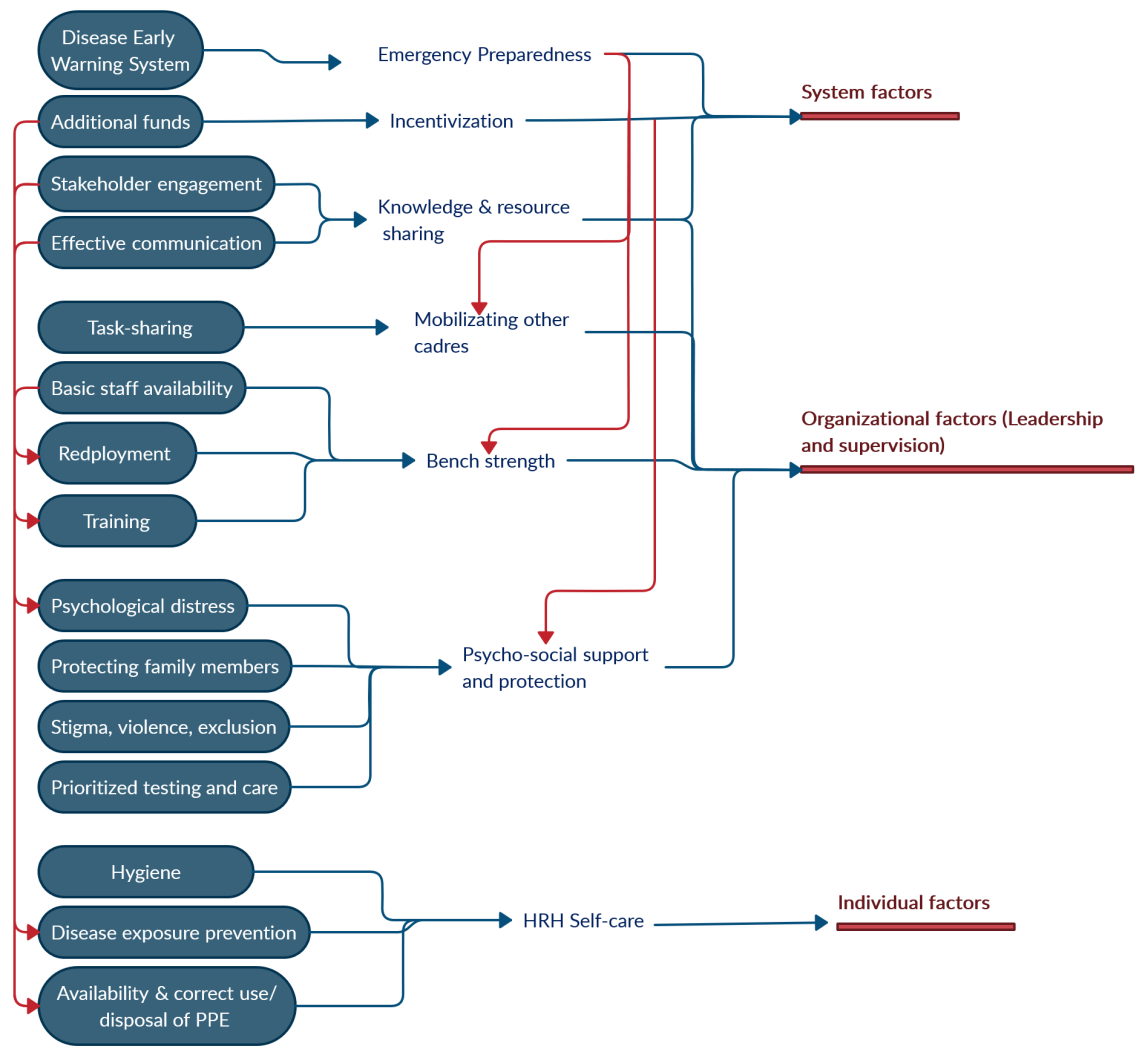
The framework highlights the challenges in a low-resource setting that could be used to inform policies for building early health system planning and preparedness for health emergencies. PPE, Personal protective equipment.

This article aimed to identify some critical areas needing policy focus that are important to consider when drafting a health workforce policy. We used a two-step process to draw the key areas for policy focus. We, first, developed a ‘HRH resilience framework’ ( Figure 1) to understand the dimensions of health workforce resilience based on a review of the present literature, understanding the existing challenges, as well as learnings, from within India and in other similar settings. We, then conducted a thematic abstraction of key challenges and identified the areas that need an immediate policy focus in order to develop a resilient workforce.

## The HRH resilience framework

Figure 1 represents an interaction of system-level, organizational and individual factors, that interact to determine the ability of the health workforce to function normally during and after a crisis.

## The challenges and efforts about the health workforce management in times of crisis

**1. The abysmally low financing of health services and a chronic shortage of skilled HRH has long defined India’s health system**

In the South-eastern region, India spends only 1.28% of GDP from the public system on health ^4^ (2017–18), while the Maldives spends about 8% (2016). In 2016, the Domestic General Government Health Expenditure per capita in the USA was $8,078 whilst this figure was a staggering $16 in India. Some of the most populous states of India spend lowest per capita. The per capita public health expenditure in Bihar (population of more than 100 million) is about $7, which is one of the lowest in the world if Bihar was a country. The poor spending on health, directly translates to a severe shortage of health workforce. India, with an estimated population of 1.32 billion, has only one government allopathic doctor for average 10,926 persons ^4^ - 10 times higher than the WHO recommendation of 1:1000 people. Furthermore, some Indian states with the poorest health index ^5^, with a predominant rural population ^6^, have only one doctor serving up to 28,000 people (EAG states) - with a higher concentration ^7^ of doctors in urban areas. Only four states have more than the recommended HRH of 44.5 per 10,000 population in India. While Delhi has the highest density of HRH of 67, Jharkhand has only 7 HRH per 10,000 persons. The low numbers are further complicated by a very skewed distribution of HRH. In 2011, only two out of five doctors were qualified in India, of which almost 75% were concentrated in urban areas ^8^ (against about 66% ^9^ Indian population residing in rural areas), and only around 10% practice in the public sector ^4^, which caters to about 30% of health care services in India. Government health facilities often fail to attract doctors due to poor quality of life, salaries, vertical growth ^10^, and job security ^11^.

**2. COVID-19 has accentuated the crisis of health service delivery, but exposure of HRH to COVID-19 worsened the situation**

HRH plays a central role in COVID-19 surveillance and management which puts them at a much higher risk of infection. In addition to the pandemic-related work, the HCW are required to continue providing routine health services, which further overburdens them. While there is a lack of clarity on the official numbers of healthcare workers (HCW) testing positive for SARS-CoV-2, unofficial figures report more than 5000 being tested positive in India by July 2020, with more than 2000 in Delhi ^12^ alone. At least 196 doctors ^13^ have already died due to infection, of which 40% were general practitioners. Presently, there is no routine testing of HCW available, and testing is limited to ‘high risk exposure’ ^14^ groups. Despite the MoHFW recommending 14-day quarantine for accidental exposure to SARS-CoV-2 for HCW, this period is often curtailed ^15^ in many Indian states. In Bihar, a deficit of health workers has forced those testing positive to continue working. Incidentally, Bihar also has a higher death rate ^16^ among doctors than other Indian states. Infection amongst HCW has severely affected the management of other diseases due to closing of hospitals ^17, 18^ and laboratories ^19^. Fear of exposure to the virus has led many doctors and nurses to avoid providing services ^20^ in private hospitals, small nursing homes and routine OPD practice at home. This indirectly heightens the burden on the public sector and increases the risk of consultations with informal providers (IP) ^21^ in rural areas- which account for 70% of all rural health service providers- who are insufficiently trained on pandemic management.

Early disease management approaches, such as recruitment of staff and facility-preparedness, has shown success in New Zealand ^22^. Also, designing a Disease early warning system (EWS) could prevent worse health outcomes (E.g. Yemen Cholera epidemic ^23^); however, it could be challenging due to COVID-19 being a respiratory illness. Additionally, having strong public-private partnerships with national and international players ^24, 25^ and involving them early in the crisis can be beneficial in planning strategies.

**3. Indian states with better workforce availability also face novel challenges that were rooted in multiple systemic constrains**

Maharashtra, one of the worst affected state, had to resource nurses and doctors from Kerala ^26^. Many states are recruiting final year medical students to meet doctor shortages. Uttar Pradesh is also facing a decline in the number of doctors due to retirement ^27^ in addition to HCW being infected. Staff deficiency has led Maharashtra to mandate private doctors to work on COVID-19, with failure to do so possibly resulting in revoking their license ^28^. The existing health workforce is constrained with additional pressure of working continuously in PPE kits, and no rest-breaks for longer working hours. Inadequate provision of PPE for sample collection and treatment ^29, 30^ had forced the HRH to wear HIV kits or raincoats and helmets. The public facility doctors ^31^ are further strained by private sector, which have the majority beds and ventilators, only handling one in 10 critical cases.

Countries such as the USA and Italy are addressing staff shortage by recruiting new HCW ^32^, medical volunteers, retired professionals, redeployment of existing HRH to different departments, focus on telemedicine and home-care ^33^. As most of the COVID-19 symptoms can be assessed virtually and do not require hospital admission, strengthening CHW and mobilizing other cadres ^34, 35^ can play a pivotal role especially in rural areas.

**4. The psycho-social impact of COVID-19 further stresses the health workforce**

A survey conducted on HCW ^36^ working directly on COVID-19 management observed higher depressive symptoms and anxiety, with women at greater risk. There were reported suicides and accidents ^37^ among HCW due to stigma and fatigue, respectively. Moreover, health workers like doctors, nurses and outreach workers have been subjected to heightened verbal and physical violence, and sexual abuse ^38^, including instances of being spat on ^39^, accusations of spreading COVID-19 in the community, and having their bags snatched ^40^. Many HCW fear infecting their families and social exclusion ^41^. Unlike past health emergencies, COVID-19 has established social media ^42^ as a perfect medium for rapid transmission of misinformation, stirring mass hysteria in the population, leading to increased attacks on HCW. The motivation of HCW is further reduced by depriving them of timely monthly salaries and implementing pay-cuts ^43, 44^. In Kerala, poor salary forced about 85% newly appointed doctors to resign ^45^.

## Key components for a health workforce resilience policy in low-resourced settings

Since India and many other countries do not have formal and comprehensive policies for health workforce augmentation, mobilization, motivation and support during extended periods of extensive shocks such as pandemics, policy frameworks to address these gaps are urgently required. Within the federal country structures, provincial policies may also be needed. The interplay of multitude rational, political and contextual factors in drafting such policies should be considered. However, there are some critical components, which almost universally form the part of HRH resilience policy. Based on the conceptual framework for HRH resilience ( Figure 1) and the challenges mentioned above, we have arrived at these components. The following components aim to address gaps of planning, staff shortage, HRH motivation, and strategic actions. These would need further expansion to draft detailed policy, nevertheless, they provide critical areas of focus for effective and efficient actions to strengthen the health workforce.

i. **Emergency preparedness planning** including early planning and building health facility readiness

ii. **Identification of bench strength of health workforce** and upskilling them (e.g. Kerala’s initial steps during COVID-19 ^46^) such as including retired workers, students, non-practicing workers, etc.

iii. **Mobilization of health workforce** from private to public sector or vice-versa, and to outbreak zones (hot spots). Redeployment of non-health community workers for health-related activities; task-sharing with trained IPs ^47^ (e.g. Liver Foundation, West Bengal).

iv. **Incentivization mechanisms** for health workforce utilized for pandemic response or exposed to risk (E.g. provision of ‘risk allowance’)

v. **Knowledge and resource sharing** by engaging stakeholders

vi. **Protection** of the HRH and their family from being infected, violence, social exclusion, and support for priority detection and treatment of illness ^48^.

vii. **Psycho-social support** for good mental health and wellbeing (online support, protective measures ^49^, and set of individual and organizational measures ^50^. Maintaining the motivation of the HRH must be prioritized. Learning from the past Ebola epidemic in Sierra Leone, provision of ‘risk allowance’ to the HRH ^51^, training/workshops including components of psycho-social support, coping strategies using social media and religious activities could be useful. Targeted interventions building motivation of the workers by addressing their concerns of pay, work-life balance, provisions for families, and timely communication of guidelines would be helpful ^52^.

viii. **HRH self-care,** including making available adequate protective gears

## Conclusions

The acute and persistent shocks on the health systems are not new, but are intensified during outbreaks such as Ebola, SARS, MERS and COVID-19. The health systems at local, national and global levels have fallen short of adequate policy needs and their execution, to remain buoyant during and after such shocks. The health workforce suffers from crises both as citizens and service providers. The increased burden and risk to health workers need to be anticipated and mitigated in advance. This certainly requires policy support at levels of the health system. The HRH resilience framework provided above, and the key components suggested for informing HRH resilience policies, would provide an important start to make further strides in this direction. We call for a multi-stakeholder approach, with a shared common objective, to frame and implement such policy, with equitable participation of the health workforce in the development of such policy. We further call for more debate and consultations to make this work further useful and contextual.

## Data availability

No data are associated with this article.
