# Supplementary material for: Impact of the COVID-19 pandemic on the human resources for health in India and key policy areas to build a resilient health workforce
Source: Gates Open Res. 2020 Oct 15;4:159. [Version 1] doi: 10.12688/gatesopenres.13196.1 (PMC7878968; doi:10.12688/gatesopenres.13196.1)
Supplement: Peer review history

# Peer review: Reviewer response for version 1

**DOI:** 10.21956/gatesopenres.14402.r30293

**Published:** 2021-02-11

**Authors:** Prashanth N Srinivas

**Affiliations:** 1 Health Equity Cluster, Institute of Public Health, Bengaluru, India

Thank you for the opportunity to review this letter. As an academic communication that seeks to critically engage with the issue of strengthening HRH from a systems lens, I appreciate the initiative by the authors in communicating this. Given that there is a systematic effort at conceiving a framework and in thematic abstraction that the authors seek to apply to guiding public policy efforts on this topic, I have reviewed it as if it were a commentary (given that this is the first time I am applying a peer review lens to a submission that is titled “open letter”) in hope that this is helpful to the authors and the audience. In terms of its readiness for publication as an open letter, I find no basis for needing significant revisions. That said, the comments below are offered to the authors as critical observations/remarks to engage with in case they move forward with the arguments presented.

**On India as an “unregulated” health system**

 The assertion (in the abstract) that India’s health system is “largely unregulated” may need critical examination. While there are huge regulatory gaps in the Indian Health System, I wonder if there is a gold-standard against which to compare/contrast regulation. This is altogether an independent area of inquiry, but without going too deep/far in that line, what I would like to point out is the need to nuance this carefully. For eg. there are aspects of Indian health services (cf. systems) which are reasonably regulated, whereas there are other aspects that are hardly even considered under formal regulatory mechanisms. Furthermore, regulation (or even lack thereof) is often a choice and hence this too ought to guide assessments of descriptions…even more so when health is a state subject and Indian states vary in terms of what aspects they choose to or do not choose to regulate.

**On the need for health workforce resilience**

 While the need for systemic resilience picks up steam in global and national policy circles, there is also a need to locate this within the wider social and political policy environment within which health policy operates. Is there sufficient demands from within communities/HRH groups for such a move? Who are the prime movers of reforms that shift systems towards greater resilience? How are these being conceived and how ought they to be conceived? These are important/useful reflections that such moves in shaping resilience literature in academia could engage with (in terms of critically examining their own positions vis-a-vis resilience building).

**On the consequences of a weak HRH capacity**

 In the section that outlines the consequences of “India’s weak HRH capacity and its inadequate deployment strategies…”, authors appear to restrict their consequences only to contentment and spread of pandemic. IT has been widely reported and analysed that this could have also impacted our ability to report numbers adequately. Indeed, states which are likely to have better-off HRH deployment did report more numbers (cf. Kerala). This could also have contributed to the overall low mortality and positive numbers from India even if it does not entirely explain this.

**On the focus on the “immediate”**

 By choosing to focus on the “immediate”, I wonder if the authors neglect possible components that could have been conceptualised only if they had envisioned a medium to long-term as well? This may be a limitation of the lens they apply.

**On the framework**

 I am a bit confused as to whether Figure 1 is a framework that they begin with or that they arrive at? (Or neither?). How did they choose which ones to include and which ones not and how did they determine the linkages? For eg. previous commitment and motivation of staff is an important input as to how they may respond to pandemics. Overall trust in public services (vs private healthcare sector) too is an important environmental determinant of how health systems perform (or don't) during pandemics but they do not figure here. And a comprehensive scoping of academic literature on resilience (or consultations with health workers themselves or with communities) may yield other relationships/linkages that are not captured here. I understand that the aim is not to develop a comprehensive framework, but figure 1’s description may have to be sharpened for it to be described as precisely as possible.

**On broadening the application of key elements of the framework**

 In the section on abysmally low financing authors pre-dominantly deal with HRH availability and appear to indicate that this is directly and probably the only(? They do not talk about any other and hence this inference). But HRH distribution and availability can be a problem even in well-financed health systems. I am not clear as to why they focus on HRH mal-distribution and availability to illustrate financing. I agree that availability may improve with financing, but it may not as well. In fact as they point out various other social and political factors, pre-dominantly at state level too determine these. Doctor/nurse/health worker unions too often safeguard their own interest and hence some of these are related to the wider political economy of availability and not only to financing in my opinion. While many of these may be outside the scope of the letter, there must be some engagement/acknowledgement of these in the narrative in my opinion.

**On public-private partnerships**

 Evidence is quite mixed in favour and/or against possible PP arrangements, certainly with “national and international players”. On the other hand policy initiatives to push forward PPPs in health have been mired not only in technical debates but also in debates related to conflicting interests, lack of accountability, poor ability of the State to safeguard the “public” in such arrangements etc. I understand again that this may be outside the scope of the letter but this will need to be engaged with critically to represent multiple views on this in my opinion.

Does the article adequately reference differing views and opinions?

Partly

Are all factual statements correct, and are statements and arguments made adequately supported by citations?

Yes

Is the Open Letter written in accessible language?

Yes

Where applicable, are recommendations and next steps explained clearly for others to follow?

Yes

Is the rationale for the Open Letter provided in sufficient detail?

Yes

Reviewer Expertise:

Health policy and systems research especially on health inequities and social determinants of health, OneHealth and intersectional action for health, evaluation of HRH interventions

I confirm that I have read this submission and believe that I have an appropriate level of expertise to confirm that it is of an acceptable scientific standard, however I have significant reservations, as outlined above.

# Peer review: Reviewer response for version 1

**DOI:** 10.21956/gatesopenres.14402.r30292

**Published:** 2021-02-08

**Authors:** Robert Mash

**Affiliations:** 1 Division of Family Medicine and Primary Care, Stellenbosch University, Cape Town, South Africa

This article presents a resilience framework for human resources for health, discusses four key issues with regard to the Indian workforce and responding to COVID-19 and finally makes a series of recommendations for the development of future policy on responding to disasters or pandemics.

 Key concepts used in the article need to be defined at the beginning or when first used. For example what is meant by resilience? What is meant by bench strength?

 The resilience framework presents 15 items that are mapped onto 7 intermediate constructs and finally three constructs at the system, organizational and individual levels. The methods and literature used to construct this framework are given only cursory attention and the rationale for the items and relationships portrayed in the framework are not elaborated. Was there a scoping review for example and if so what are the details of this? Explain the framework to the reader. For the reader to accept the validity of the framework and to understand it, both of these issues need to be tackled.

 It is said that the literature was from India and similar settings, however the other countries mentioned are New Zealand, USA and Italy. I am not sure that these can be considered similar settings.

 The four key issues that are discussed are said to be a ‘thematic abstraction’ from this framework. What exactly this means is not clear. The conceptual links to the framework are not explained and the framework itself and the four key issues appear to be unrelated to each other and to a large extent stand-alone from each other in the article.

 The final list of recommendations for policy are interesting and probably make sense. However they do not obviously all flow from the four key issues and the resilience framework. Again there is a disconnect for the reader. As far as I can understand it the logical flow of the article is (1) a resilience framework for HRH was constructed from the literature. (2) key issues were abstracted from this framework (3) policy recommendations were derived from these key issues. The linkages between these components and the logical flow need to be more explicit.

 There are numerous grammatical and linguistic issues in the article which I have made note of in this file.

Does the article adequately reference differing views and opinions?

Partly

Are all factual statements correct, and are statements and arguments made adequately supported by citations?

No

Is the Open Letter written in accessible language?

Partly

Where applicable, are recommendations and next steps explained clearly for others to follow?

Partly

Is the rationale for the Open Letter provided in sufficient detail?

Yes

Reviewer Expertise:

Family medicine, primary health care, health services and chronic diseases.

I confirm that I have read this submission and believe that I have an appropriate level of expertise to confirm that it is of an acceptable scientific standard, however I have significant reservations, as outlined above.

# Peer review: Reviewer response for version 1

**DOI:** 10.21956/gatesopenres.14402.r30044

**Published:** 2020-12-03

**Authors:** Giorgio Cometto

**Affiliations:** 1 Health Workforce Department, World Health Organization, Geneva, Switzerland

I would like to congratulate the authors for this interesting submission. I would encourage the authors to provide additional details if available on the results/impact of the policy recommendations made. 

 I would also encourage them to review and consider the recommendations in the WHO guidance on health workforce policy and management in the context of COVID-19, which adopts a similar framework and provides many similar policy recommendations.

 Another useful reference to consider in your bibliography is the similarly related paper (but taking a global overview) by Ivy Bourgeault in the HRH Journal ^1^. But very supportive of this manuscript being indexed.

Does the article adequately reference differing views and opinions?

Yes

Are all factual statements correct, and are statements and arguments made adequately supported by citations?

Yes

Is the Open Letter written in accessible language?

Yes

Where applicable, are recommendations and next steps explained clearly for others to follow?

Yes

Is the rationale for the Open Letter provided in sufficient detail?

Yes

Reviewer Expertise:

Health systems - human resources for health

I confirm that I have read this submission and believe that I have an appropriate level of expertise to confirm that it is of an acceptable scientific standard.

## References

[1] : The COVID-19 pandemic presents an opportunity to develop more sustainable health workforces. *Human Resources for Health*.2020;18(1) : 10.1186/s12960-020-00529-0 10.1186/s12960-020-00529-0 33129313PMC7602762
